# Differences in cardiometabolic risk profiles between Chinese and Finnish older adults with glucose impairment and central obesity

**DOI:** 10.1007/s40618-022-01777-8

**Published:** 2022-03-24

**Authors:** S. Le, Y. Zhang, A. Voutilainen, X. Tan, J. Laukkanen, C. Wang, S. Cheng

**Affiliations:** 1 Exercise Translational Medicine Center, Shanghai Center for Systems Biomedicine, Shanghai Jiao Tong University, 200240 Shanghai, China; 2 Key Laboratory of Systems Biomedicine (Ministry of Education), Shanghai Center for Systems Biomedicine, Shanghai Jiao Tong University, Shanghai, 200240 China; 3 Department of Physical Therapy, Taihe Hospital, Hubei University of Medicine, Shiyan, 442099 China; 4 Faculty of Sport and Health Sciences, University of Jyväskylä, 40014 Jyväskylä, Finland; 5 Shanghai Jiao Tong University Affiliated Sixth People’s Hospital, The Metabolic Disease Biobank, Shanghai, 200233 China; 6 Institute of Public Health and Clinical Nutrition, University of Eastern Finland, 70211 Kuopio, Finland; 7 Department of Neuroscience, Uppsala University, BMC, Box 593, 75124 Uppsala, Sweden; 8 Institute of Clinical Medicine, Department of Medicine, University of Eastern Finland, 70211 Kuopio, Finland; 9 Department of Endocrinology and Metabolism, School of Medicine, Shanghai Fourth People’s Hospital Affiliated to Tongji University, Tongji University, 1279 Sanmen Road, Shanghai, 200434 China

**Keywords:** Type 2 diabetes mellitus, Chinese, Finnish, Ethnicity, Central obesity, Cardiovascular disease risk

## Abstract

**Background:**

Obesity and ethnicity play important roles in cardiovascular complications in patients with type 2 diabetes mellitus (T2DM). This study aimed to compare cardiometabolic risk profiles between Chinese and Finnish older adults of central obesity with prediabetes or T2DM.

**Methods:**

Study subjects were 60–74 years old and originated from two population samples. The Finnish subjects came from the Kuopio Ischemic Heart Disease (KIHD) study (*n* = 1089), and the Chinese subjects came from the Shanghai High-risk Diabetic Screen (SHiDS) study (*n* = 818). The KIHD and SHiDS studies used similar questionnaires to determine participants’ baseline characteristics regarding the history of medication use and diseases and lifestyle factors. All study subjects participated in glucose tolerance tests and anthropometry assessments, including waist circumference measurements.

**Results:**

Among study subjects of central obesity with prediabetes (*n* = 298), fasting and 2-h glucose, and fasting insulin and insulin resistance were significantly higher in Chinese than in Finnish (*p* < 0.0001–0.016). In addition, triglyceride (TG) level was higher and the low-density lipoprotein cholesterol (LDL) and LDL to high-density lipoprotein cholesterol (HDL) ratio were lower in Chinese than in Finnish (*p* < 0.0001–0.003). Among subjects of central obesity with T2DM (*n* = 251), Chinese subjects had significantly less proportions of antihypertensive, glycaemic control medication, and statin users as well as lower level of physical activity (*p* < 0.0001 for all), while higher blood pressure (*p* = 0.002 for systolic blood pressure and *p* < 0.0001 for diastolic blood pressure), TG levels (*p* < 0.05) and HDL (*p* = 0.002) than the Finnish counterparts. There were no differences in β-cell function (HOMA-β) between Chinese and Finnish both in prediabetes and T2DM.

**Conclusions:**

Our results indicated that Chinese and Finnish older adults of central obesity with prediabetes and T2DM had similar β-cell function. However, Chinese individuals with prediabetes are prone to insulin resistance. Meanwhile, lipid metabolism dysfunction is also different between Chinese and Finnish. Chinese older adults of central obesity with prediabetes showed higher TG, but Finnish showed higher LDL and LDL/HDL. Strategic for T2DM prevention and treatment should be ethnically specific.

## Introduction

Type 2 diabetes mellitus (T2DM) is the most common metabolic disease, with an increasing prevalence worldwide [1, 2]. According to the 2019 International Diabetes Federation report, the estimated total number of adults with diabetes is 463.0 million worldwide, and among them, more than 90% have T2DM [1]. Of note, the highest estimated prevalence of diabetes is in people older than 65, with an estimated number of individuals with diabetes of 135.6 million in 2019, and this number is expected to increase dramatically to 276.2 million by 2045 [1]. Cardiovascular diseases (CVDs) are a major cause of death and disability in patients with T2DM [3, 4]. More than 50% of all patients with T2DM die of CVDs [5]. In elderly individuals with T2DM, a higher absolute risk of CVDs combined with geriatric conditions leads to a high mortality rate and increased hospital admission and institutionalization, which comes with inevitable costs to public health and economic challenges [6].

Ethnicity is an important factor that needs to be considered for T2DM and diabetes-related complications. For some ethnic groups, there is a greater predisposition for developing T2DM and diabetes-related complications [5, 7–12]. Compared to their European counterparts, East Asian patients have a greater tendency to develop T2DM at a younger age due to β cell dysfunction and insulin resistance [13, 14]. On the other hand, compared to Asian individuals, European individuals with diabetes are at higher risk of complications such as myocardial infarction, coronary artery disease, lower-extremity gangrene, and large vessel disease but have a lower risk of small vessel disease and diabetic eye and kidney diseases [5, 9, 10]. This predisposition of different ethnic groups to T2DM and its complications are commonly attributed to the complex interaction of genetic and environmental factors [15, 16].

Previous studies have shown that cardiovascular biomarkers, low-density lipoprotein (LDL), total cholesterol (TC), cardiac troponin T and C-reactive protein are systematically higher in Caucasian diabetic patients with acute coronary syndrome than in their Chinese counterparts [17]. Ethnic variations in cardiometabolic risk factors contribute at least in part to the excess CVDs in ethnic minorities. In addition, obesity, especially central obesity, positively associated with increased prevalence of CVDs in T2DM and prediabetes [18–25]. Using the primary care database of the UK, one study showed that there are important distinctions between ethnic groups in the relationship between obesity and CVDs, with South Asians showing greater susceptibility to CVDs even at lower body mass index (BMI) levels [26]. However, it is still unclear whether the differences in risk factors between Caucasian and Chinese individuals with prediabetes and T2DM are associated with central obesity.

While obesity and ethnicity play important roles in cardiovascular complications in patients with T2DM, there is almost no any study that has evaluated ethnicity-specific cardiovascular risk profiles of adults with both glucose impairment and central obesity at the population level. In the context of increasingly ethnically diverse individuals and ageing in many countries, especially European countries, studies on the relationship between ethnicity, obesity, and cardiovascular complications in elderly individuals with glucose impairment could have an immense impact on public health and the socioeconomic burden of CVDs associated with T2DM [27–29]. Thus, the objective of this study was to compare the cardiometabolic risk profiles between Chinese and Finnish older adults of central obesity with glucose impairment (prediabetes or T2DM). We hypothesized that there are differences in certain risk factors between Chinese and Finnish due to ethnical background, genetics and lifestyle. At the given BMI, Chinese would more disposed to insulin resistance than the Finnish whom with glucose impairment.

## Subjects and methods

### Study design and participants

The study subjects were derived from two well-defined population samples. The Finnish subjects came from the Kuopio Ischemic Heart Disease (KIHD) study [30], and the Chinese subjects came from the Shanghai High-risk Diabetic Screen (SHiDS) study [31]. The Finnish KIHD study was a prospective population-based cohort study designed to investigate risk factors for atherosclerotic CVD and other related chronic disease outcomes. The KIHD cohort was employed for the current analysis and initially comprised 1774 invited participants (920 women and 854 men) who were aged 53–74 years who participated from March 1998 to February 2001. The Chinese study was conducted at the 6th Hospital affiliated with Shanghai Jiao Tong University. The total sample consisted of 2445 Chinese individuals, with an age range from 30 to 74. Since we are interested in late adulthood, only those aged 60 ≤ to ≤ 74 years were included in this report. Thus, the final sample comprised 1089 Finnish and 818 Chinese individuals.

Similar questionnaires examining history of medication use and diseases; lifestyle, including smoking, drinking and physical activity habits; and education level were used to characterize the background of the participants. The Finnish study was approved by the Ethics Committee of Kuopio University and Kuopio University Hospital, Kuopio, Finland (licence number 143/97), and the Chinese study was approved by the Ethics Committee of Shanghai Jiao Tong University 6^th^ Hospital (2018-KY-056 (K)). Written informed consent was obtained from all participants, and all study procedures were conducted according to the Declaration of Helsinki.

### Anthropometric and body composition assessment

Body weight and height were measured with standard methods and used to calculate BMI. Blood pressure was measured with the conventional method (in Finland with a Hawksley random zero sphygmomanometer and in China with an Omron HBP-9020 s (Omron Healthcare, Kyoto, Japan)). Waist circumference (WC) and hip circumference were measured by tape [32]. The waist circumference to hip circumference ratio (WHR) was calculated.

### Biochemical measurements

Blood samples were collected in the morning between 7:00 and 9:00 a.m. after overnight fasting at both sites. Serum was extracted by centrifugation and stored immediately at  – 80 °C until analysis. Glucose tolerance tests were performed at both sites after overnight fasting, and samples were taken 30 min and 2 h after the intake of 75 g glucose for the assessment of serum insulin (chemiluminescent immunoassay) and glucose (automatic biochemistry analyser). The homeostasis model assessment of insulin resistance index (HOMA-IR) was calculated as (fasting insulin concentration × fasting glucose concentration)/22.5, and the β-cell function index (HOMA-β) was calculated as (20 × fasting insulin concentration/(fasting glucose concentration  – 3.5)[33]. The serum lipid [34] and plasma glucose [30] analysis methods used in Finland have been described previously. In China, the plasma glucose analysis method has been described previously [31], and serum TC, high-density lipoprotein (HDL), LDL and triglyceride (TG) were analysed using a Hitachi Automatic Analyzer 7600 (Hitachi High-Technologies, Tokyo, Japan). The inter- and intra-assay CVs were 3.4% and 2.9% for TG, 2.0% and 3.7% for glucose, and 11% and 3.4% for insulin, respectively.

### Criteria for prediabetes, T2DM and central obesity

The criterion for prediabetes was defined according to the American Diabetes Association criteria as a fasting glucose level from 5.6 mmol/l to 6.9 mmol/l or an OGTT 2 h glucose level from 7.0 mmol/l to 11.0 mmol/l [35]. The criterion for T2DM was defined as a fasting glucose level ≥ 7.0 mmol/l or an OGTT 2 h glucose level ≥ 11.1 mmol/l [35]. Subjects receiving drug therapy for diabetes were also included in the T2DM group. Central obesity was indicated by waist circumference ≥ 94 cm for Europid men, ≥ 90 cm for Chinese men and ≥ 80 cm for women in both countries [36].

### Statistical analysis

Statistical analyses were performed using IBM SPSS Statistics for Windows, Version 21.0. Descriptive statistics were generated and presented for each ethnic group, including percentages for categorical variables and means and 95% confidence intervals for continuous variables. The Shapiro–Wilk test was used to check all data for normality. If the data were not normally distributed, a log transformation was used to ensure a normal distribution. Categorical variables were compared between Chinese and Finnish individuals using the chi-square test for independence. Continuous variables were compared between Chinese and Finnish individuals by using Student’s *t*-test in univariate analysis or Mann–Whitney *U* tests. Analysis of covariance (ANCOVA) was used to control for confounding factors such as age, sex, BMI and medicine when comparing the variables between the Chinese and Finnish populations. Pearson correlation analysis was performed to evaluate the correlation between central obesity (BMI and waist circumference) and glycaemic and lipid variables. In addition, generalized linear models (GLMs) were used to control for confounding factors such as age, sex, BMI and medicine when assessing the relationships among BMI, WC and glycaemic and lipid variables.

## Results

### Profiles of Chinese and Finnish older adults with glucose impairment and central obesity

Among the targeted population, 18.3% (*n* = 199) of the Finnish population had prediabetes, and 7.9% (*n* = 85) had T2DM, while 30.6% (*n* = 250) had prediabetes and 31.8% (*n* = 260) had T2DM in the Chinese population. Since the purpose of this report was to examine the differences in the cardiometabolic risk profiles of Finnish and Chinese older adults with glucose impairment and central obesity, the following results are only related to the comparison of prediabetes and T2DM between Finnish and Chinese individuals with central obesity.

The general characteristics of the subjects with prediabetes and T2DM and central obesity were shown in Table 1. Finnish individuals were older (*p* < 0.0001 for all) and heavier (*p* < 0.0001 for all) and had a larger BMI (*p* < 0.0001 for all), but higher participation in physical activity (*p* < 0.0001 for all) than Chinese individuals in both the prediabetes and T2DM groups.

**Table 1:** General characteristics of Chinese and Finnish older adults with glucose impairment and central obesity

	Pre-diabetes				T2DM			
	Chinese		Finnish		Chinese		Finnish	
	*n*	Mean (95% CI)	*n*	Mean (95% CI)	*n*	Mean (95% CI)	*n*	Mean (95% CI)
Age, years	147	63.7 (63.2, 64.1)	151	67.9 (67.2, 68.5)^**^	172	64.4 (63.9, 64.9)	79	67.9 (67, 68.7 ^**^
Height, cm	147	162 (161, 163)	151	165 (164, 167)^##^	172	164 (162, 165)	79	164 (162, 167)
Weight, kg	147	67.2 (65.4, 69.0)	151	82.0 (80.2, 83.7)^##^	172	68.5 (66.9, 70)	79	80.8 (77.8, 83.8) ^##^
BMI, kg/m^2^	147	25.5 (25, 26)	151	30.1 (29.5, 30.6)^##^	172	25.5 (25.1, 25.9)	79	29.8 (29, 30.6) ^##^
WC, cm	147	91.5 (90.4, 92.7)	151	99.0 (97.7, 100.5)^**^	172	92.9 (91.8,94.1)	79	100.4 (98.1,102.7) ^**^
WHR	146	0.92 (0.91, 0.93)	151	0.94 (0.92, 0.95)	172	0.94 (0.93, 0.95)	79	0.96 (0.94, 0.98) ^#^
SBP, mmHg	146	139 (136, 142)	151	141 (138, 144)	170	137 (135, 140)	79	133 (129, 137) ^#††^
DBP, mmHg	146	81.6 (80.1, 83.1)	151	81.1 (79.7, 82.6)	170	82.4 (81.0, 83.9)	79	75.8 (73.7, 77.8) ^##††^
Education, 1/2/3^a^, %^†^	146	6.2/73.3/20.5	151	80.8/15.2/4.0 ^##^	172	5.2/61.0/33.7	79	87.3/10.1/2.5 ^##^
Gender (female/male), %^†^	147	68.7/31.3	151	51.7/48.3 ^##^	172	66.9/33.1	79	49.4/50.6 ^##^
Smoking (current/former/never), %^†^	145	4.1/13.0/82.9	151	10.6/27.8/61.6 ^##^	164	10.4/9.4/80.2	76	7.9/28.5/63.6 ^##^
Alcohol drinking (yes/no), %^†^	146	17.8/82.2	151	6.0/94.0 ^##^	164	10.4/89.6	77	13.0/87.0
Diabetes of family (yes/no), %^†^	143	38.5/61.5	151	37.7/62.3	165	51.5/48.5	79	48.1/51.9
Use of statins (yes/no), %^†^	147	1.4/98.6	151	5.3/94.7	172	4.1/95.9	79	13.9/86.1 ^##^
Use of antihypertensive medication (yes/no), %^†^	147	9.5/90.5	151	58.9/41.1 ^##^	172	30.8/69.2	79	78.5/21.5 ^##^
Use of antidiabetic medication (yes/no), %^†^	NA	NA	NA	NA	172	1.7/98.3	48	60.8/39.2 ^##^
Physical activity (yes/no), %^†^	146	77.4/22.6	151	98.7/1.3 ^##^	172	72.7/27.3	77	96.1/3.9 ^##^
Frequency, times/week	113	4.6 (4.0, 5.2)	151	6.7 (6.0, 7.4)^**^	99	5.6 (4.9, 6.3)	73	5.4 (4.8, 6.1)
Duration, hours/week	113	4.6 (3.8, 5.4)	151	10.2 (8.7, 11.4)^**^	99	5.1 (4.3, 5.9)	73	7.8 (6.5, 9.2)^**^
Intensity (low/medium/high), %	113	53.1/46.0/0.9	149	6.0/90.6/3.4 ^##^	125	82.4/17.6/0.0	74	12.2/78.4/9.5 ^##^

*CI* confidence interval, *T2DM* type 2 diabetes, *BMI* body mass index, *WC* waist circumference, *WHR* waist-to-hip ratio, *SBP* systolic blood pressure, *DBP* diastolic blood pressure, *NA* not applicable

BMI in T2DM was log-transformed for the analysis

^a^Education: 1 = primary/below, 2 = high school, 3 = graduate/above

^†^Values are number of subjects with proportion (%)

^*^p < 0.05, **p < 0.01, comparison of the means between Chinese and Finns by the Mann–Whitney *U* test

^#^p < 0.05, ## p < 0.01, comparison of the means between Chinese and Finns by Student’s t-test or proportions by chi-square test or rank sum test

^††^p < 0.01, comparison of the means between Chinese and Finn populations by analysis of covariance (ANCOVA) controlling for age, sex, BMI and anti-hypertension medicine

Among prediabetes patients, the proportion of alcohol drinking was significantly higher in Chinese individuals (*p* = 0.002), but lower in smoking (*p* < 0.0001), using antihypertensive medication (*p* < 0.0001) than their Finnish counterparts (*p* = 0.033).

Among T2DM patients, the Chinese population had a smaller WHR (*p* = 0.046), lower using antihypertensive and antidiabetic medication as well as statins, but higher blood pressure (*p* = 0.04 for SBP and *p* < 0.0001 for diastolic blood pressure (DBP)) than the Finnish population, and after controlling for age, sex, BMI, and the use of antihypertensive medication, the significance remained the same.

**Fig. 1:**
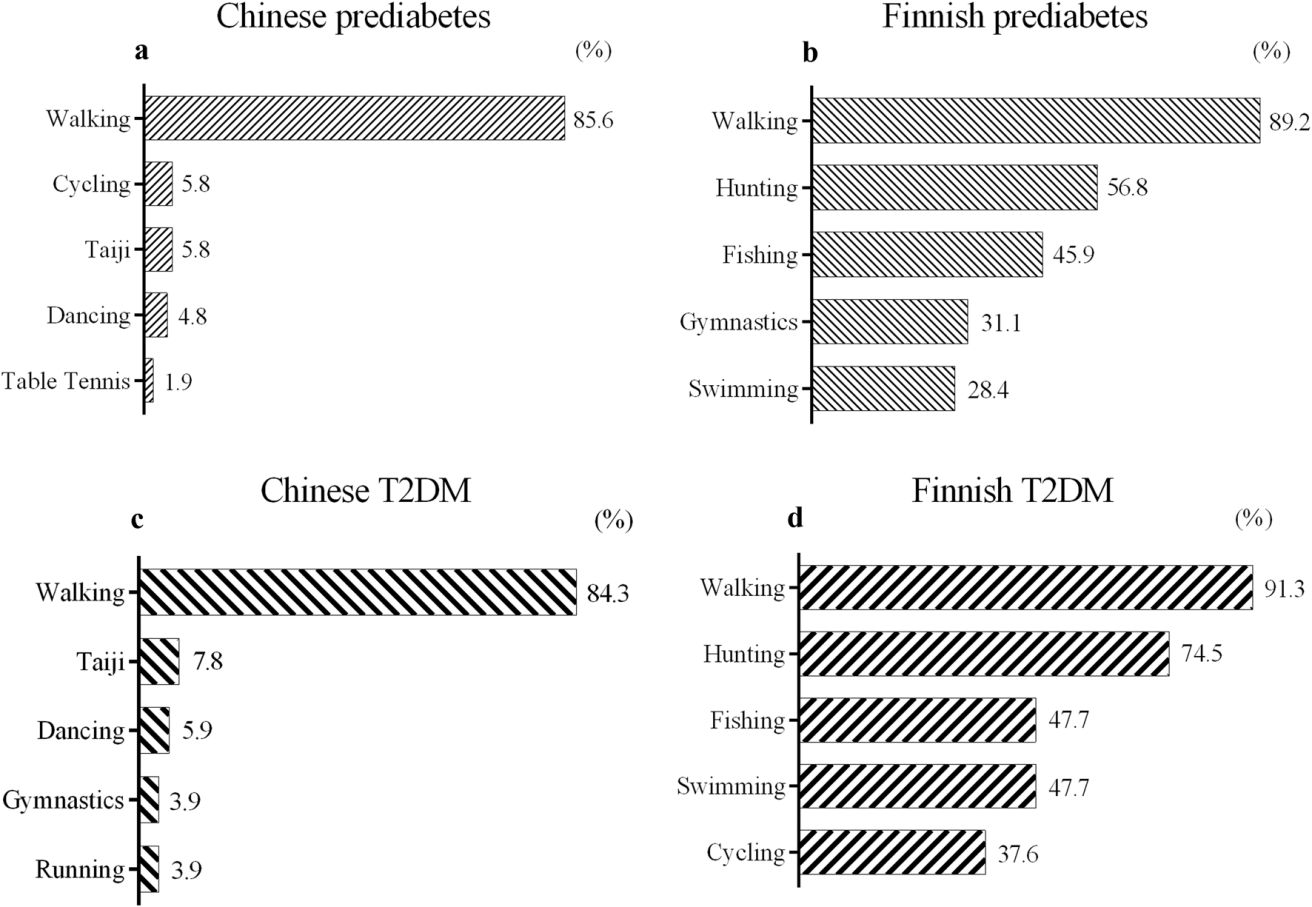
Top 5 LTPAs participated by Chinese and Finnish with glucose impairment and central obesity. *LTPA* leisure time physical activities

Walking was the most common type of leisure time physical activity (LTPA) for both Finnish and Chinese individuals. In addition to walking, hunting, fishing, and gymnastics were common LTPAs among Finnish individuals, while less than 10% of Chinese engaged in Taiji, dancing, and jogging (Fig. 1). Among prediabetes patients, Chinese individuals reported participating in LTPA less frequently (*p* = 0.003), for a shorter duration (*p* < 0.0001) and at a lower intensity (*p* < 0.0001) than Finnish individuals. In T2DM patients, Chinese patients had a similar frequency of LTPA to Finnish patients but a shorter duration (*p* = 0.002) and lower intensity (*p* < 0.0001).

For the purpose of comparison, we also assessed the differences between subjects without and with central obesity. The results are given in Supplementary Tables 1 and 2.

### Comparison of glycaemic variables between Chinese and Finnish older adults with glucose impairment and central obesity

Among prediabetes patients, the Chinese population had higher fasting glucose (*p* < 0.0001) and 2 h glucose (*p* = 0.016) (see Table 2). After controlling for age, sex and BMI, Chinese individuals had higher fasting insulin (adjusted mean difference of the log-transformed = 0.167, 95% CI: 0.102, 0.232, df = 1, F = 25.643, p < 0.0001, partial η^2^ = 0.082) and HOMA-IR (adjusted mean difference of the log-transformed = 0.204, 95% CI 0.136, 0.271, df = 1, F = 35.088, *p* < 0.0001, partial η^2^ = 0.109). After additional confounding factors, such as antidiabetic medication, SBP, education, physical activity, smoking and alcohol consumption, were added to the model, the significance remained the same.

**Table 2:** Glycaemic variables of Chinese and Finnish older adults with glucose impairment and central obesity

	Pre-diabetes				T2DM			
	Chinese		Finnish		Chinese		Finnish	
	*n*	Mean (95% CI)	*n*	Mean (95% CI)	*n*	Mean (95% CI)	*n*	Mean (95% CI)
Glu_0_, mmol/l	147	5.85 (5.75, 5.95)	151	5.42 (5.32, 5.53) ^**^	172	6.97 (6.79, 7.14)	79	7.94 (7.35, 8.53)
Glu_2h_, mmol/l	147	8.29 (8.01, 8.58)	151	7.96 (7.73, 8.2) ^*^	172	14.8 (14.3, 15.2)	57	14.5 (13.1, 16) ^#^
Ins_0_, mmol/l	143	12.9 (11.7, 14.1)	151	11.2 (10.4, 12.1) ^##††^	171	13.5 (12.4, 14.5)	79	16.9 (12.7, 21)
HOMA-IR	143	3.33 (3.02, 3.64)	150	2.73 (2.49, 2.97) ^##††^	170	4.13 (3.79, 4.46)	79	6.45 (4.21, 8.7)
HOMA-β	137	111 (99, 123)	150	125 (115, 135)	170	85.6 (77.5, 93.7)	79	93.4 (76.5, 110.3)

*T2DM* type 2 diabetes, *CI* confidence interval, *Glu*_*0*_ fasting glucose level, *Glu*_*2h*_ 2-h glucose level, *Ins*_*0*_ fasting insulin level, *HOMA-IR* homeostasis model assessment of insulin resistance, *HOMA-β* homeostasis model assessment of β-cell function index

Ins_0_, HOMA-IR and HOMA-β in prediabetes and Glu_0_, Glu_2h_, Ins_0_, and HOMA-IR in T2DM were log-transformed for the analysis

^*^p < 0.05, **p < 0.01, comparison of the means between Chinese and Finnish populations by the Mann–Whitney *U* test

^#^*p* < 0.05, ##*p* < 0.01, comparison of the means between Chinese and Finnish populations by analysis of covariance (ANCOVA) controlling for age, sex, BMI and anti-diabetes medicine

^††^*p* < 0.01, comparison of the means between Chinese and Finnish individuals by ANCOVA controlling for age, sex, BMI, anti-diabetes medicine, SBP, education, physical activity, smoking and alcohol drinking

In T2DM patients, after controlling for age, sex, BMI and use of antidiabetic medication, 2-h glucose was significantly higher in Chinese individuals than in Finnish individuals (*p* = 0.043). However, after adding SBP, education, LTPA, smoking and alcohol drinking to the model, this significance disappeared.

### Comparison of lipid profiles between Chinese and Finnish older adults with glucose impairment and central obesity

**Fig. 2:**
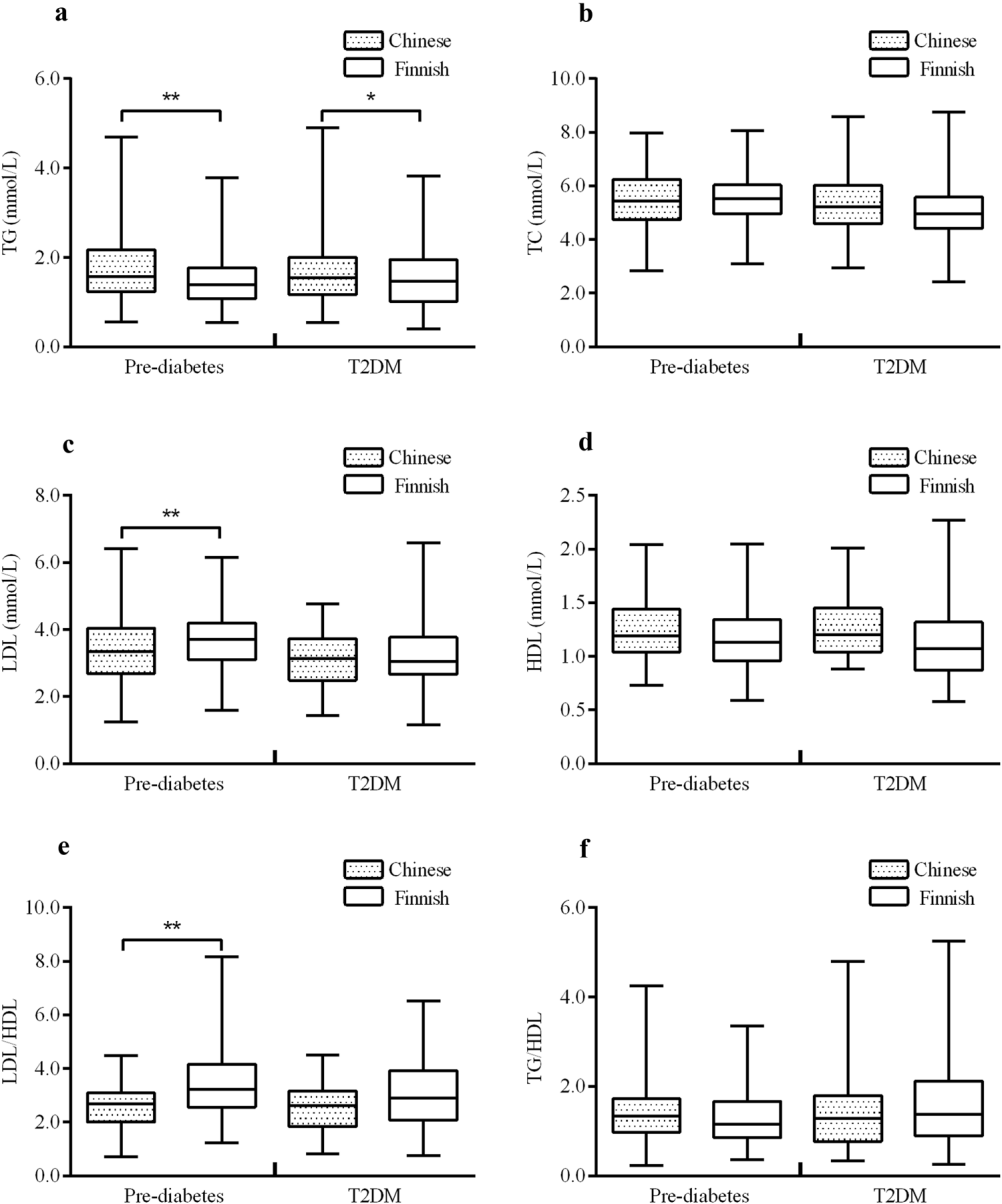
Comparison of lipid profiles between Chinese and Finnish populations with glucose impairment and central obesity. *TG* triglyceride, *TC* total cholesterol, *LDL* low-density lipoprotein cholesterol, *HDL* high-density lipoprotein cholesterol. The Mann–Whitney *U* test was used to assess the difference in HDL in between Chinese and Finnish T2DM populations. Analysis of covariance (ANCOVA) was used to assess the differences in the remaining variables adjusting for age, sex, BMI and use of statins. *p < 0.05; **p < 0.01

In prediabetes patients, after controlling for age, sex, BMI, and the use of statins, the Chinese population had higher TG (adjusted mean difference = 0.475, 95% CI 0.205, 0.746, df = 1, F = 11.955, *p* = 0.001) but lower LDL (adjusted mean difference =  – 0.522, 95% CI  – 0.864,  – 0.180, df = 1, F = 9.029, *p* = 0.003) levels and LDL/HDL ratios (adjusted mean difference =  – 0.759, 95% CI  – 1.130,  – 0.389, df = 1, F = 16.271, *p* < 0.0001, Fig. 2).

In T2DM patients, only TG levels were higher in Chinese patients (adjusted mean difference = 0.412, 95% CI 0.055, 0.769, df = 1, F = 5.193, *p* = 0.024). No other differences between the Chinese and Finnish populations were observed.

### Correlations between central obesity and glycaemic and lipid profiles

We assessed the correlations between central obesity and glycaemic and lipid profiles. The results are given in Supplementary Table 3. Significant associations were found between BMI/WC and insulin/HOMA-IR in both the Finnish and Chinese populations (all *p* < 0.05–0.001). In addition, in Finnish individuals, WC was significantly correlated with fasting glucose, TG, HDL and TG/HDL in prediabetes (all *p* < 0.05–0.0001), while TG and TG/HDL ratio were positively correlated with BMI and WC, and HDL was negatively correlated with WC in T2DM (all *p* < 0.05–0.0001). In Chinese individuals, BMI and WC was positively correlated with HOMA-β (both *p *= 0.0001) and WC with TG/HDL ratio (*p* = 0.042) in prediabetes patients and BMI with HOMA-β in T2DM patients (*p* = 0.012), respectively. Controlling for all the confounding factors (age, sex, drinking, smoking, medications and LTPA), the significant associations between WC and HDL in Finnish T2DM and WC and TG/HDL ratio in Chinese and Finnish pre-diabetes disappeared. Interestingly, the associations between HOMA-β and BMI/WC in Finnish individuals and between HOMA-β and WC in Chinese T2DM appeared to be significant.

In addition, we assessed the correlations between glycaemic and lipid profiles. The results are given in Supplementary Table 4. Significant associations were found between TG levels and TG/HDL ratio and insulin/HOMA-IR/HOMA-β in both the Finnish and Chinese prediabetes (all *p* < 0.05–0.001). However, HDL was correlated with HOMA-β only in Chinese pre-diabetes but not in Finnish. In T2DM, TG levels were correlated with fasting insulin level and HOMA-IR and TG/HLD ratio with 2 h glucose level, fasting insulin level and HOMA-IR only in Finnish T2DM not in Chinese, while HDL was correlated with fasting glucose and 2 h glucose level in Chinese not in Finnish. Controlling for all the confounding factors (age, sex, drinking, smoking, medications and LTPA), the associations in prediabetes and in Finnish T2DM were still significant, while the significant associations between HDL and fasting glucose and 2 h glucose level in Chinese T2DM disappeared.

## Discussion

In this cross-sectional study, we compared the cardiometabolic profiles of Finnish and Chinese older adults with prediabetes or T2DM and central obesity, and we observed several differences between these two ethnic groups. Specifically, among older adult participants with prediabetes and central obesity, fasting glucose, insulin, HOMA-IR, and TG were significantly higher, while LDL and LDL/HDL ratio were lower, in Chinese individuals than in Finnish individuals. Even after controlling for all confounding factors, significance remained. However, among older adult participants with T2DM and central obesity, only TG was significantly higher in Chinese individuals than in Finnish individuals. Interestingly, the associations between BMI/WC and HOMA-IR/HOMA-β were similar in the prediabetes and T2DM populations and between ethnic groups.

Most of the early reports on the differences in risk factors for prediabetes and T2DM between ethnicities did not take central obesity into account. In this study, we found that the differences between subjects with and without central obesity were mainly in weight, WC, insulin level and insulin resistance in both ethnic groups and in both the prediabetes and T2DM populations. This result indicated that in addition to central obesity, insulin resistance is one of the first signs of glucose impairment in the development of T2DM regardless of ethnicity. On the other hand, prediabetes and T2DM in Chinese individuals may be due more to dysfunction of the cardiovascular system, as indicated by blood pressure, than in Finnish individuals. One meta-analysis showed that the prevalence of overall CVDs, stroke and coronary artery disease was higher in Western Pacific (including China) than in European T2DM populations [5].

When only subjects with central obesity and prediabetes were included, the differences between Finnish and Chinese subjects were related not only to insulin resistance but also to the levels of glucose, TG and LDL and the LDL/HLD ratio. Central obesity rather than obesity defined by BMI has been associated with various detrimental metabolic consequences, such as dysglycaemia and dyslipidaemia, all of which cause cardiovascular mortality [37]. A consensus statement pointed out that [37] “the lack of inclusion of waist circumference in global obesity surveillance might inadequately characterize the health risk associated with the global obesity prevalence, as it seems that the prevalence of abdominal obesity is increasing”; therefore, it was recommended that measurement of waist circumference should be included in obesity surveillance studies. In this study, we included measures of WC and WHR. We found that in prediabetes patients, there were significant differences in both BMI and WC but not in WHR between Finnish and Chinese individuals. Higher BMI and WC were significantly correlated with higher levels of fasting insulin and HOMA-IR/HOMA-β in both ethnic groups. These associations were independent of age, sex, drinking, smoking, medications and physical activity indicating that fatness is a dominant factor that impact on insulin resistance and function regardless of age, sex and lifestyle factors.

However, there are ethnic differences regarding lipid metabolism between Finnish and Chinese individuals with prediabetes or T2DM. Compared with the Finnish, Chinese older adults of central obesity with glucose impairment had higher TG levels. The risk of unhealthy lipid profiles, such as high TG, may lead to cardiovascular diseases [38], dementia caused by Alzheimer’s disease [39], and frailty [40], thus predisposing patients to an increased risk of mortality and lower quality of life. It is possible that differences in body composition, particularly visceral fat accumulation, cause high TG in Asian populations [41]. A previous study showed that compared with European cohorts, Chinese cohorts had a relatively greater amount of abdominal adipose tissue, and this difference was more pronounced in visceral fat [42]. Greater visceral fat mass was also associated with both higher TG levels and higher insulin resistance levels in normal weight but metabolically obese Japanese adults [43]. However, in this report, we did not have body composition data; thus, it cannot be confirmed that the high TG levels in Chinese older adults are associated with high body fat content. On the other hand, we noticed that the Chinese population in both the prediabetes and T2DM groups spent much less time performing LTPAs (mean difference: 5.52 and 2.72 h/week, separately) and did so at a lower intensity (more than 50% low intensity) than their Finnish counterparts. Low levels of PA or a sedentary lifestyle may lead to high TG levels and insulin resistance [44–46]. Therefore, promotion of physical activity in the Chinese population of central obesity with prediabetes or T2DM is a necessary strategy for prevention.

Existing studies have mainly compared the differences between different ethnic groups within the same country, and fewer comparisons of the differences between ethnic groups on different continents have been reported [11, 47, 48]. Increased mobility due to globalization, particularly during the past two decades, has brought new challenges for health care systems in different countries. Immigrants frequently take their culture and lifestyle with them to their new place of settlement [49]. There are differences in predisposition to developing T2DM and diabetes-related complications [5, 7–11]. However, there are no ethnically specific criteria or strategic considerations for T2DM prevention, diagnosis and treatment in clinical practice in any country. Our results provide information on risk factor differences from countries on different continents. However, this study has some limitations. The study has a cross-sectional design. The Chinese T2DM patients were mostly newly diagnosed, which may have influenced the outcomes. No body composition measurements were taken, no dietary information was collected, and differences in dietary intake may have impacted the results, since glycaemic responses following ingestion of glucose and several rice varieties are appreciably greater in Chinese individuals than in European individuals [50]. Two cohorts were significantly different in BMI and age, which are major confounding factors of cardiometabolic profiles, making difficult to compare two different cohorts. To make the two cohorts more comparable, we adjusted these two confounders factor together with other confounder factors, the main significant results remained the same. In addition, we selected subgroups of participants with similar age and BMI, performed the same analysis, and found the same results as with adjusting the confounder factors (date are not shown). Therefore, the differences found in cardiometabolic profiles between the two cohorts are independent of BMI and age in this study. Nevertheless, this study may encourage clinicians to take into account the differences between ethnic groups in the future.

## Conclusions

In conclusion, by comparing the cardiometabolic profiles of Finnish and Chinese older adults of central obesity with prediabetes and T2DM, we found that both Finnish and Chinese had similar β-cell function. However, Chinese individuals with prediabetes are prone to insulin resistance. Meanwhile, lipid metabolism dysfunction is also different between Chinese and Finnish prediabetes. Chinese individuals with prediabetes showed higher TG, but Finnish showed higher LDL and LDL/HDL. Strategic for T2DM prevention and treatment should be ethnically specific.

## Data Availability

The data that support the findings of this study are available from the corresponding author upon reasonable request.

## Abbreviations

T2DM: Type 2 diabetes mellitus
KIHD: Kuopio ischemic heart disease
SHiDS: Shanghai high-risk diabetic screen
TG: Triglyceride
HOMA-IR: Homeostasis model assessment of insulin resistance index
LDL: Low-density lipoprotein cholesterol
HDL: High-density lipoprotein cholesterol
CVDs: Cardiovascular diseases
TC: Total cholesterol
BMI: Body mass index
WC: Waist circumference
WHR: Waist circumference to hip circumference
HOMA-β: Homeostasis model assessment of β-cell function index
ANCOVA: Analysis of covariance
GLM: Generalized linear model
SBP: Systolic blood pressure
DBP: Diastolic blood pressure
LTPA: Leisure time physical activity
CI: Confidence interval
NA: Not applicable
Glu0: Fasting glucose level
Glu2h: 2-H glucose level
Ins0: Fasting insulin level

## References

[1] International Diabetes Federation. IDF Diabetes Atlas 2019. 2019. https://diabetesatlas.org/en/ resources/IDF Diabetes Atlas 2019. Accessed 8 Mar 2021. 35914061

[2] World Health Organization. Global report on diabetes. 2016. https://www.who.int/publications/i/item/ 9789241565257. Accessed 8 Mar 2021.

[3] International Diabetes Federation (2016). Diabetes and cardiovascular disease.

[4] Jankauskas SS, Kansakar U, Varzideh F, Wilson S, Mone P, Lombardi A (2021). Heart failure in diabetes. Metabolism.

[5] Einarson TR, Acs A, Ludwig C (2018). Prevalence of cardiovascular disease in type 2 diabetes: a systematic literature review of scientific evidence from across the world in 2007–2017. Cardiovasc Diabetol.

[6] Sinclair A, Morley JE, Rodriguez-Mañas L, Paolisso G, Bayer T, Zeyfang A (2012). Diabetes mellitus in older people: position statement on behalf of the International Association of Gerontology and Geriatrics (IAGG), the European Diabetes Working Party for Older People (EDWPOP), and the International Task Force of Experts in Diabetes. J Am Med Dir Assoc.

[7] Paul SK, Owusu Adjah ES, Samanta M, Patel K, Bellary S, Hanif W (2017). Comparison of body mass index at diagnosis of diabetes in a multi-ethnic population: A case-control study with matched non-diabetic controls. Diabetes Obes Metab.

[8] Hsu WC, Araneta MR, Kanaya AM, Chiang JL, Fujimoto W (2015). BMI cut points to identify at-risk Asian Americans for type 2 diabetes screening. Diabetes Care.

[9] Ma RC, Chan JC (2013). Type 2 diabetes in East Asians: similarities and differences with populations in Europe and the United States. Ann N Y Acad Sci.

[10] Zheng Y, Ley SH, Hu FB (2018). Global aetiology and epidemiology of type 2 diabetes mellitus and its complications. Nat Rev Endocrinol.

[11] Meeks KAC, Freitas-Da-Silva D, Adeyemo A, Beune EJAJ, Modesti PA, Stronks K (2016). Disparities in type 2 diabetes prevalence among ethnic minority groups resident in Europe: a systematic review and meta-analysis. Intern Emerg Med.

[12] Anjana RM, Lakshminarayanan S, Deepa M, Farooq S, Pradeepa R, Mohan V (2009). Parental history of type 2 diabetes mellitus, metabolic syndrome, and cardiometabolic risk factors in Asian Indian adolescents. Metabolism.

[13] Chan JCN, Malik V, Jia W, Kadowaki T, Yajnik CS, Yoon K-H (2009). Diabetes in Asia: epidemiology, risk factors, and pathophysiology. JAMA.

[14] Kato N (2012). Ethnic diversity in type 2 diabetes genetics between East Asians and Europeans. J Diabetes Investig.

[15] Carulli L, Rondinella S, Lombardini S, Canedi I, Loria P, Carulli N (2005). Review article: diabetes, genetics and ethnicity. Aliment Pharmacol Ther.

[16] Murea M, Ma L, Freedman BI (2012). Genetic and environmental factors associated with type 2 diabetes and diabetic vascular complications. Rev Diabet Stud.

[17] Buljubasic N, Zhao W, Cheng J, Li H, Oemrawsingh R, Akkerhuis M (2020). Comparison of temporal changes in established cardiovascular biomarkers after acute coronary syndrome between Caucasian and Chinese patients with diabetes mellitus. Biomarkers.

[18] Katsiki N, Anagnostis P, Kotsa K, Goulis DG, Mikhailidis DP (2019). Obesity, metabolic syndrome and the risk of microvascular complications in patients with diabetes mellitus. Curr Pharm Des.

[19] Boonman-de Winter LJ, Rutten FH, Cramer MJ, Landman MJ, Liem AH, Rutten GE (2012). High prevalence of previously unknown heart failure and left ventricular dysfunction in patients with type 2 diabetes. Diabetologia.

[20] Bhatti GK, Bhadada SK, Vijayvergiya R, Mastana SS, Bhatti JS (2016). Metabolic syndrome and risk of major coronary events among the urban diabetic patients: North Indian Diabetes and Cardiovascular Disease Study-NIDCVD-2. J Diabetes Complic.

[21] Wentworth JM, Fourlanos S, Colman PG (2012). Body mass index correlates with ischemic heart disease and albuminuria in long-standing type 2 diabetes. Diabetes Res Clin Pract.

[22] Glogner S, Rosengren A, Olsson M, Gudbjörnsdottir S, Svensson AM, Lind M (2014). The association between BMI and hospitalization for heart failure in 83,021 persons with Type 2 diabetes: a population-based study from the Swedish National Diabetes Registry. Diabet Med.

[23] Laitinen T, Lindström J, Eriksson J, Ilanne-Parikka P, Aunola S, Keinänen-Kiukaanniemi S (2011). Cardiovascular autonomic dysfunction is associated with central obesity in persons with impaired glucose tolerance. Diabet Med.

[24] Di Bonito P, Pacifico L, Chiesa C, Valerio G, Miraglia Del Giudice E, Maffeis C (2017). Impaired fasting glucose and impaired glucose tolerance in children and adolescents with overweight/obesity. J Endocrinol Invest.

[25] Czernichow S, Kengne A-P, Huxley RR, Batty GD, de Galan B, Grobbee D (2011). Comparison of waist-to-hip ratio and other obesity indices as predictors of cardiovascular disease risk in people with type-2 diabetes: a prospective cohort study from ADVANCE. Eur J Cardiovasc Prev Rehabil.

[26] Owusu Adjah ES, Bellary S, Hanif W, Patel K, Khunti K, Paul SK (2018). Prevalence and incidence of complications at diagnosis of T2DM and during follow-up by BMI and ethnicity: a matched case-control analysis. Cardiovasc Diabetol.

[27] Gholap N, Davies M, Patel K, Sattar N, Khunti K (2011). Type 2 diabetes and cardiovascular disease in South Asians. Prim Care Diabetes.

[28] Bodicoat DH, Gray LJ, Henson J, Webb D, Guru A, Misra A (2014). Body mass index and waist circumference cut-points in multi-ethnic populations from the UK and India: the ADDITION-Leicester, Jaipur heart watch and New Delhi cross-sectional studies. PLoS ONE.

[29] Fernando E, Razak F, Lear SA, Anand SS (2015). Cardiovascular disease in South Asian migrants. Can J Cardiol.

[30] Kunutsor SK, Laukkanen JA (2016). Serum zinc concentrations and incident hypertension: new findings from a population-based cohort study. J Hypertens.

[31] Gao F, Zhang Y, Ge S, Lu H, Chen R, Fang P (2018). Coffee consumption is positively related to insulin secretion in the Shanghai High-Risk Diabetic Screen (SHiDS) Study. Nutr Metab.

[32] Millar SR, Perry IJ, Van den Broeck J, Phillips CM (2015). Optimal central obesity measurement site for assessing cardiometabolic and type 2 diabetes risk in middle-aged adults. PLoS ONE.

[33] Matthews DR, Hosker JP, Rudenski AS, Naylor BA, Treacher DF, Turner RC (1985). Homeostasis model assessment: insulin resistance and β-cell function from fasting plasma glucose and insulin concentrations in man. Diabetologia.

[34] Salonen JT, Salonen R, Seppanen K, Rauramaa R, Tuomilehto J (1991). HDL, HDL2, and HDL3 subfractions, and the risk of acute myocardial infarction. A prospective population study in eastern Finnish men. Circulation.

[35] American Diabetes Association (2019). 2 Classification and Diagnosis of Diabetes: Standards of Medical Care in Diabetes 2019. Diabetes Care.

[36] Alberti KG, Zimmet P, Shaw J (2006). Metabolic syndrome–a new world-wide definition. a consensus statement from the international diabetes federation. Diabet Med.

[37] Ross R, Neeland IJ, Yamashita S, Shai I, Seidell J, Magni P (2020). Waist circumference as a vital sign in clinical practice: a Consensus Statement from the IAS and ICCR Working Group on Visceral Obesity. Nat Rev Endocrinol.

[38] Nordestgaard BG, Varbo A (2014). Triglycerides and cardiovascular disease. Lancet.

[39] Raffaitin C, Gin H, Empana JP, Helmer C, Berr C, Tzourio C (2009). Metabolic syndrome and risk for incident Alzheimer's disease or vascular dementia: the Three-City Study. Diabetes Care.

[40] Ramsay SE, Arianayagam DS, Whincup PH, Lennon LT, Cryer J, Papacosta AO (2015). Cardiovascular risk profile and frailty in a population-based study of older British men. Heart.

[41] Hwang YC, Fujimoto WY, Hayashi T, Kahn SE, Leonetti DL, Boyko EJ (2016). Increased visceral adipose tissue is an independent predictor for future development of atherogenic dyslipidemia. J Clin Endocrinol Metab.

[42] Lear SA, Humphries KH, Kohli S, Chockalingam A, Frohlich JJ, Birmingham CL (2007). Visceral adipose tissue accumulation differs according to ethnic background: results of the Multicultural Community Health Assessment Trial (M-CHAT). Am J Clin Nutr.

[43] Katsuki A, Sumida Y, Urakawa H, Gabazza EC, Murashima S, Maruyama N (2003). Increased visceral fat and serum levels of triglyceride are associated with insulin resistance in Japanese metabolically obese, normal weight subjects with normal glucose tolerance. Diabetes Care.

[44] Bailey DP, Charman SJ, Ploetz T, Savory LA, Kerr CJ (2017). Associations between prolonged sedentary time and breaks in sedentary time with cardiometabolic risk in 10–14-year-old children: The HAPPY study. J Sports Sci.

[45] Fernández-García JC, Muñoz-Garach A, Martínez-González MÁ, Salas-Salvado J, Corella D, Hernáez Á (2020). Association between lifestyle and hypertriglyceridemic waist phenotype in the PREDIMED-plus study. Obesity.

[46] Dos Santos ESM, Máximo RO, de Andrade FB (2021). Differences in the prevalence of prediabetes, undiagnosed diabetes and diagnosed diabetes and associated factors in cohorts of Brazilian and English older adults. Public Health Nutr.

[47] Zafarmand MH, Tajik P, Spijker R, Agyemang C (2020). Gene-environment Interaction on the Risk of Type 2 Diabetes Among Ethnic Minority Populations Living in Europe and North America: A Systematic Review. Curr Diabetes Rev.

[48] Dal Canto E, Farukh B, Faconti L (2018). Why are there ethnic differences in cardio-metabolic risk factors and cardiovascular diseases?. JRSM Cardiovasc Dis.

[49] Popovic-Lipovac A, Strasser B (2015). A review on changes in food habits among immigrant women and implications for health. J Immigr Minor Health.

[50] Kataoka M, Venn BJ, Williams SM, Te Morenga LA, Heemels IM, Mann JI (2013). Glycaemic responses to glucose and rice in people of Chinese and European ethnicity. Diabet Med.

